# Supplementary material for: The Association between Blood Indexes and Immune Cell Concentrations in the Primary Tumor Microenvironment Predicting Survival of Immunotherapy in Gastric Cancer
Source: Cancers (Basel). 2022 Jul 25;14(15):3608. doi: 10.3390/cancers14153608 (PMC9332606; doi:10.3390/cancers14153608)
Supplement: Supplementary file 1 [file cancers-1804145-supplementary.pdf › cancers-1804145-supplementary.pdf]

Original Article

The association between blood indexes and immune cell concentrations in the primary tumor microenvironment predicting survival of immunotherapy in gastric cancer

Jiajia Yuan[1†], Xingwang Zhao[1†], Yanyan Li[1], Qian Yao[2], Lei Jiang[1], Xujiao Feng[1], Lin Shen[1], Yilin Li[1*] and Yang Chen[1*]

[1] Department of Gastrointestinal Oncology, Key Laboratory of Carcinogenesis and Translational Research (Ministry of Education), Peking University Cancer Hospital and Institute, Beijing, 100142, China; jyuan@bjmu.edu.cn (JJ.Y.), qiuyesiyu3@163.com (YY.L.), zhaoxingwang237@163.com (XW.Z), ncuskjianglei@163.com (L.J.), 13293656798@163.com (XJ.F.), shenlin@bjmu.edu.cn (L.S.),

[2] Department of Pathology, Key Laboratory of Carcinogenesis and Translational Research (Ministry of Education), Peking University Cancer Hospital and Institute, Beijing, 100142, China; yaoqian@bjmu.edu.cn (Y.Q.)

† These authors contributed equally to this work.

***** Correspondence: liyilin@bjcancer.org (YL.L), Yang_chen@bjcancer.org (Y.C.); Tel.: 010-88196747(Y.L. &Y.C.)

Supplementary Table S1. Antibodies used for mIHC

CD8a (Cell Signaling Technology, Cat. CST70306)
LAG-3 (Cell Signaling Technology, Cat. CST15372)
TIM-3 (Cell Signaling Technology, Cat. CST45208)
PD-1 (Cell Signaling Technology, Cat. CST43248)
CD4 (Biolynx, Cat. BX22300130)
PD-L1 (Cell Signaling Technology, Cat. CST13684)
CTLA-4 (Abcam, Cat. ab227709)
FoxP3 (Biolegend, Cat. BLG320202)
CD68 (ZSGB-BIO, Cat. ZM0060)
CD163 (Cell Signaling Technology, Cat. CST93498)
HLA-DR (Abcam, Cat. ab92511)
STING (Cell Signaling Technology, Cat. CST13647)
CD20 (Abcam, Cat. ab78237)
CD66b (Gene Tex, Cat. GTX19779)
CD147 (Cell Signaling Technology, Cat. CST13287)
CLDN18.2 (Abcam, Cat. ab22512)
PanCK (Cell Signaling Technology, Cat. CST4545)

Supplementary Table S2. Association between immune markers and immune cell density in the tumor microenvironment of tumor core

Variables	CD8$^+$	CD8$^+$LAG3$^+$PD1$^+$	CD8$^+$LAG3$^+$PD1$^-$	CD8$^+$LAG3$^-$PD1+	CD8$^+$LAG3$^-$PD1$^-$	CD8$^+$PD1$^+$TIM3$^+$	CD8$^+$PD1$^-$TIM3$^+$	CD8$^+$LAG3$^+$PD1$^+$TIM3$^+$	CD8$^+$LAG3$^+$PD1$^+$TIM3$^-$	CD8$^+$LAG3$^-$PD1$^+$TIM3$^+$	CD8$^+$LAG3$^-$PD1$^+$TIM3$^-$	CD4$^+$	CD4$^+$FOXP3$^+$	CD4$^+$FOXP3$^-$	CD4$^+$CTLA4$^+$FOXP3$^+$	CD4$^+$CTLA4$^+$FOXP3$^-$	CD4$^+$FOXP3$^+$PDL1$^+$	CD4$^+$FOXP3$^-$PDL1$^+$	CD68$^+$	CD68$^+$STING$^+$	CD163$^+$CD68$^+$HLADR$^-$	CD163$^-$CD68$^+$HLADR$^+$	CD163$^+$CD68$^+$HLADR$^-$STING$^+$	CD163$^-$CD68$^+$HLADR$^+$STING$^+$	CD20$^+$	CD66B$^+$	CLDN18.2$^+$	CD147$^+$
Neut (*p*-value)	0.041	0.094	0.038	0.257	0.040	0.235	0.015	0.434	0.014	0.212	0.379	0.165	0.685	0.069	0.974	0.115	0.755	0.193	0.057	0.019	0.347	0.046	0.053	0.049	0.336	0.115	0.228	0.177
Low	311.2	10.0	8.1	83.3	209.8	16.0	19.8	5.2	4.8	10.8	72.5	567.6	136.1	431.5	5.2	37.9	47.8	198.9	845.4	87.5	21.9	308.2	2.4	25.1	216.4	525.0	2504.8	3416.7
High	468.1	18.5	19.3	109.2	321.0	27.9	56.4	7.6	10.9	20.3	88.9	726.7	125.9	600.8	5.1	70.0	51.4	272.3	1114.9	166.6	31.5	418.8	8.6	44.8	300.0	707.9	2973.2	3941.6
Mono (*p*-value)	0.042	0.130	0.014	0.180	0.054	0.107	0.014	0.351	0.052	0.081	0.352	0.247	0.553	0.208	0.118	0.140	0.653	0.289	0.051	0.279	0.484	0.271	0.350	0.374	0.012	0.023	0.160	0.426
Low	315.4	10.6	7.4	81.7	215.7	14.4	20.5	5.0	5.5	9.3	72.4	583.9	123.9	460.0	3.3	39.7	47.1	207.1	848.7	109.4	23.3	334.2	4.1	30.6	156.5	491.7	2479.7	3531.5
High	471.7	18.3	20.6	112.3	320.4	30.4	57.6	7.9	10.4	22.5	89.9	716.9	138.8	578.1	7.3	69.8	52.4	267.1	1125.3	146.5	30.5	395.9	7.1	39.7	370.6	754.3	3025.5	3842.3
Lymph (*p*-value)	0.746	0.913	0.405	0.479	0.494	0.715	0.101	0.931	0.746	0.657	0.295	0.744	0.952	0.702	0.421	0.640	0.713	0.923	0.972	0.691	0.337	0.916	0.980	0.340	0.645	0.141	0.509	0.850
Low	376.7	14.6	11.4	104.6	246.2	20.1	25.3	6.3	8.3	13.8	90.8	627.9	130.2	497.7	6.3	49.1	47.4	232.8	982.7	120.0	21.7	366.5	5.5	30.0	237.6	528.6	2606.9	3641.2
High	401.9	14.0	15.9	88.4	283.7	23.7	50.3	6.5	7.5	17.2	71.1	665.4	131.7	533.7	4.2	58.6	51.7	238.3	977.6	133.7	31.5	360.6	5.6	39.6	277.8	699.9	2864.6	3715.3
Plt (*p*-value)	0.253	0.927	0.113	0.861	0.166	0.648	0.037	0.963	0.896	0.561	0.983	0.192	0.470	0.161	0.973	0.129	0.295	0.282	0.039	0.183	0.099	0.047	0.448	0.128	0.398	0.142	0.210	0.358
Low	345.3	14.0	9.4	94.3	227.6	19.7	22.3	6.3	7.7	13.3	80.9	572.4	121.9	450.5	5.2	38.5	43.5	205.2	834.0	104.3	18.4	308.3	4.3	27.2	221.4	531.0	2495.3	3500.1
High	434.0	14.5	18.0	98.3	303.2	24.2	53.9	6.5	8.0	17.8	80.5	721.8	140.0	581.7	5.1	69.4	55.7	266.0	1126.3	149.8	35.1	418.7	6.8	42.6	295.0	701.9	2982.7	3858.2
NLR (*p*-value)	0.029	0.015	0.011	0.039	0.081	0.050	0.010	0.079	0.005	0.062	0.080	0.158	0.575	0.059	0.793	0.275	0.853	0.173	0.131	0.119	0.531	0.225	0.089	0.222	0.576	0.371	0.154	0.350
Low	306.0	8.1	6.9	73.0	218.1	12.3	18.7	3.7	4.4	8.5	64.4	566.2	138.0	428.2	5.5	42.8	48.5	197.2	872.7	100.4	23.5	329.6	2.8	28.8	233.8	564.2	2462.5	3497.1
High	473.3	20.4	20.5	119.6	312.7	31.7	57.5	9.0	11.3	22.6	97.0	728.0	124.0	604.1	4.8	65.1	50.7	274.0	1087.5	153.6	29.9	397.4	8.3	41.1	282.6	668.6	3015.4	3861.2
LMR (*p*-value)	0.096	0.045	0.023	0.107	0.205	0.101	0.031	0.203	0.012	0.100	0.193	0.581	0.881	0.474	0.287	0.438	0.902	0.488	0.062	0.452	0.512	0.616	0.503	0.883	0.876	0.055	0.343	0.539
Low	453.8	19.3	19.8	114.7	300.0	30.1	54.4	8.3	11.0	21.8	92.9	678.8	129.1	549.8	6.6	61.9	48.9	255.2	1112.4	139.9	30.1	377.6	6.6	35.7	265.0	727.5	2923.7	3799.1
High	325.5	9.2	7.6	77.9	230.9	13.8	21.8	4.5	4.7	9.4	68.5	615.4	132.9	482.5	3.8	46.0	50.3	216.0	847.8	114.1	23.4	349.4	4.4	34.2	251.4	505.4	2554.3	3559.2
PLR (*p*-value)	0.007	0.268	0.022	0.016	0.014	0.107	0.004	0.324	0.292	0.085	0.026	0.041	0.264	0.027	0.628	0.082	0.124	0.065	0.004	0.161	0.131	0.001	0.467	0.260	0.318	0.423	0.700	0.585
Low	287.8	11.4	7.5	69.1	199.6	14.0	16.8	4.9	6.5	9.1	60.1	530.7	117.0	413.6	4.6	36.3	40.7	183.9	778.8	103.1	19.1	276.6	4.3	29.2	214.8	569.6	2663.8	3572.6
High	491.5	17.1	19.8	123.4	331.2	30.0	59.5	7.9	9.2	22.1	101.3	763.5	144.9	618.6	5.8	71.6	58.5	287.3	1181.4	151.0	34.4	450.5	6.7	40.6	301.6	663.2	2814.2	3785.7
SII (*p*-value)	0.047	0.482	0.029	0.198	0.046	0.272	0.012	0.648	0.383	0.207	0.289	0.110	0.673	0.065	0.810	0.062	0.301	0.197	0.010	0.010	0.235	0.002	0.052	0.041	0.584	0.098	0.413	0.553
Low	313.3	12.5	7.8	81.6	211.4	16.5	19.3	5.7	6.8	10.8	70.8	555.8	125.7	430.1	4.9	35.1	43.6	199.2	798.4	83.8	20.7	279.0	2.4	24.7	234.3	520.5	2579.3	3563.4

High	466.0	16.1	19.6	111.0	319.4	27.4	56.9	7.1	9.0	20.4	90.6	738.4	136.3	602.2	5.5	72.9	55.6	272.0	1161.9	170.3	32.8	448.1	8.7	45.2	282.1	712.3	2898.6	3794.8

Supplementary Table S3 Association between immune markers and immune cell density in the tumor microenvironment in tumor region

Variables	CD8^{+}	CD8^{+}LAG3^{+}PD1^{+}	CD8^{+}LAG3^{+}PD1^{-}	CD8^{+}LAG3^{-}PD1+	CD8^{+}LAG3^{-}PD1^{-}	CD8^{+}PD1^{+}TIM3^{+}	CD8^{+}PD1^{-}TIM3^{+}	CD8^{+}LAG3^{+}PD1^{+}TIM3^{+}	CD8^{+}LAG3^{+}PD1^{+}TIM3^{-}	CD8^{+}LAG3^{-}PD1^{+}TIM3^{+}	CD8^{+}LAG3^{-}PD1^{+}TIM3^{-}	CD4^{+}	CD4^{+}FOXP3^{+}	CD4^{+}FOXP3^{-}	CD4^{+}CTLA4^{+}FOXP3^{+}	CD4^{+}CTLA4^{+}FOXP3^{-}	CD4^{+}FOXP3^{+}PDL1^{+}	CD4^{+}FOXP3^{-}PDL1^{+}	CD68^{+}	CD68^{+}STING^{+}	CD163^{+}CD68^{+}HLADR^{-}	CD163^{-}CD68^{+}HLADR^{+}	CD163^{+}CD68^{+}HLADR^{-}STING^{+}	CD163^{-}CD68^{+}HLADR^{+}STING^{+}	CD20^{+}	CD66B^{+}	CLDN18.2^{+}	CD147^{+}
Neut (*p*-value)	0.155	0.198	0.069	0.758	0.136	0.620	0.026	0.710	0.034	0.565	0.834	0.328	0.605	0.171	0.814	0.208	0.583	0.127	0.044	0.043	0.215	0.024	0.182	0.035	0.068	0.100	0.336	0.600
Low	206.1	9.7	6.8	60.1	129.5	12.1	13.4	5.2	4.5	6.9	53.2	358.1	89.5	268.6	3.7	25.5	32.4	130.2	800.9	61.8	15.0	293.0	1.8	19.3	108.0	459.6	3748.5	4464.5
High	302.3	15.9	15.3	65.3	205.8	15.1	35.3	6.3	9.6	8.8	56.4	458.2	77.6	380.7	4.3	50.7	38.1	209.9	1173.6	135.7	33.1	466.8	11.4	42.8	225.9	672.6	4311.2	4701.9
Mono (*p*-value)	0.090	0.376	0.033	0.594	0.075	0.398	0.018	0.634	0.238	0.263	0.736	0.082	0.107	0.087	0.057	0.094	0.168	0.175	0.004	0.271	0.681	0.015	0.718	0.124	0.012	0.046	0.216	0.878
Low	199.9	10.8	6.3	58.4	124.4	11.1	13.3	5.1	5.7	6.0	52.3	324.0	65.9	258.1	1.5	22.2	28.6	136.3	739.0	79.5	26.9	291.3	7.8	22.8	90.6	443.9	3686.8	4550.2
High	314.2	15.0	16.3	67.4	215.5	16.3	36.5	6.5	8.5	9.8	57.6	501.2	103.0	398.2	6.8	55.7	42.6	207.3	1261.7	120.0	20.8	477.9	5.2	40.2	251.3	701.2	4409.0	4619.7
Lymph (*p*-value)	0.864	0.715	0.781	0.163	0.812	0.997	0.134	0.966	0.438	0.976	0.128	0.783	0.877	0.764	0.567	0.627	0.472	0.565	0.895	0.719	0.846	0.977	0.461	0.415	0.817	0.147	0.447	0.909
Low	260.2	13.7	10.4	74.7	161.4	13.6	16.7	5.7	8.0	7.9	66.8	393.7	81.7	312.0	4.8	33.1	31.5	154.5	974.5	91.9	25.5	378.7	9.3	26.3	174.7	469.7	3801.8	4556.5
High	248.5	11.9	11.7	51.2	173.6	13.6	31.6	5.8	6.1	7.8	43.4	422.0	85.3	336.7	3.2	42.9	38.8	184.8	999.3	105.2	22.6	381.0	4.0	35.6	159.6	657.8	4246.7	4608.5
Plt (*p*-value)	0.900	0.524	0.299	0.202	0.830	0.776	0.085	0.808	0.331	0.761	0.184	0.528	0.972	0.437	0.731	0.321	0.375	0.280	0.052	0.666	0.838	0.056	0.597	0.245	0.084	0.108	0.305	0.496
Low	258.5	14.3	8.6	73.4	162.1	14.5	15.8	6.1	8.2	8.3	65.0	375.8	83.1	292.7	4.5	28.1	30.7	141.7	807.3	90.8	22.5	305.9	8.5	24.5	111.1	462.0	3730.4	4429.4
High	249.9	11.3	13.5	51.9	173.2	12.7	32.8	5.4	5.9	7.3	44.6	440.6	83.9	356.6	3.5	48.1	39.8	198.4	1167.2	106.7	25.5	453.9	4.7	37.6	222.8	670.2	4329.2	4737.0
NLR (*p*-value)	0.083	0.038	0.006	0.207	0.153	0.169	0.006	0.169	0.015	0.193	0.276	0.467	0.389	0.249	0.874	0.494	0.646	0.128	0.148	0.316	0.321	0.274	0.198	0.495	0.088	0.315	0.247	0.794
Low	195.8	7.9	4.7	52.0	131.1	9.4	11.0	3.7	4.1	5.6	46.4	370.9	93.5	277.4	4.2	31.2	32.9	130.3	852.4	80.3	16.7	337.3	2.0	27.2	111.7	500.7	3692.3	4524.0
High	312.6	17.7	17.4	73.3	204.2	17.8	37.7	7.8	10.0	10.0	63.2	445.5	73.6	371.9	3.8	45.0	37.6	209.7	1122.1	117.2	31.3	422.5	11.2	34.9	222.2	631.5	4367.4	4642.4
LMR (*p*-value)	0.061	0.061	0.011	0.171	0.108	0.209	0.012	0.297	0.014	0.170	0.232	0.325	0.665	0.268	0.189	0.259	0.550	0.230	0.019	0.748	0.656	0.200	0.652	0.796	0.171	0.064	0.533	0.488
Low	317.2	17.3	17.0	74.2	208.7	17.4	36.7	7.3	10.0	10.2	64.0	458.6	88.5	370.1	5.8	49.4	38.3	201.5	1204.2	104.7	20.7	429.8	5.0	32.5	211.4	685.8	4212.2	4740.0
High	191.2	8.3	5.2	51.1	126.6	9.7	12.0	4.2	4.1	5.5	45.6	357.8	78.5	279.2	2.2	26.8	32.2	138.6	770.3	92.8	27.3	330.0	8.2	29.6	122.5	446.4	3847.4	4426.4
PLR (*p*-value)	0.092	0.666	0.057	0.174	0.119	0.546	0.033	0.657	0.752	0.477	0.184	0.201	0.832	0.124	0.619	0.183	0.175	0.085	0.005	0.498	0.926	0.001	0.607	0.182	0.020	0.514	0.906	0.698
Low	197.4	11.8	6.6	51.2	127.8	11.7	13.8	5.1	6.7	6.6	44.6	342.9	81.1	261.8	3.3	24.7	28.4	125.2	730.3	86.2	23.3	251.9	8.5	23.5	92.1	523.6	4064.4	4671.1
High	311.0	13.8	15.5	74.1	207.5	15.4	34.8	6.4	7.4	9.0	65.0	473.5	86.0	387.5	4.7	51.5	42.2	214.9	1244.2	111.2	24.7	508.0	4.7	38.6	241.8	608.6	3995.2	4495.3
SII (*p*-value)	0.396	0.981	0.064	0.988	0.338	0.963	0.047	0.964	0.993	0.901	0.965	0.313	0.851	0.189	0.813	0.167	0.343	0.149	0.015	0.038	0.159	0.002	0.178	0.030	0.104	0.127	0.565	0.880
Low	225.4	12.9	6.7	62.8	143.0	13.4	14.6	5.8	7.0	7.6	55.2	356.6	85.7	270.9	3.7	24.3	30.4	132.3	763.3	60.9	13.7	261.7	1.8	18.9	114.3	467.3	3861.4	4617.3
High	283.0	12.7	15.4	62.5	192.3	13.7	34.1	5.7	7.1	8.0	54.5	459.8	81.4	378.5	4.3	51.9	40.1	207.8	1211.2	136.6	34.3	498.1	11.4	43.2	219.6	664.9	4198.3	4549.1

Supplementary Table S4 Association between immune markers and immune cell density in the tumor microenvironment in stromal region

Variables	CD8^{+}	CD8^{+}LAG3^{+}PD1^{+}	CD8^{+}LAG3^{+}PD1^{-}	CD8^{+}LAG3^{-}PD1+	CD8^{+}LAG3^{-}PD1^{-}	CD8^{+}PD1^{+}TIM3^{+}	CD8^{+}PD1^{-}TIM3^{+}	CD8^{+}LAG3^{+}PD1^{+}TIM3^{+}	CD8^{+}LAG3^{+}PD1^{+}TIM3^{-}	CD8^{+}LAG3^{-}PD1^{+}TIM3^{+}	CD8^{+}LAG3^{-}PD1^{+}TIM3^{-}	CD4^{+}	CD4^{+}FOXP3^{+}	CD4^{+}FOXP3^{-}	CD4^{+}CTLA4^{+}FOXP3^{+}	CD4^{+}CTLA4^{+}FOXP3^{-}	CD4^{+}FOXP3^{+}PDL1^{+}	CD4^{+}FOXP3^{-}PDL1^{+}	CD68^{+}	CD68^{+}STING^{+}	CD163^{+}CD68^{+}HLADR^{-}	CD163^{-}CD68^{+}HLADR^{+}	CD163^{+}CD68^{+}HLADR^{-}STING^{+}	CD163^{-}CD68^{+}HLADR^{+}STING^{+}	CD20^{+}	CD66B^{+}	CLDN18.2^{+}	CD147^{+}
Neut (*p*-value)	0.028	0.079	0.065	0.132	0.031	0.298	0.039	0.347	0.025	0.310	0.162	0.092	0.947	0.043	0.720	0.095	0.832	0.242	0.117	0.053	0.440	0.278	0.048	0.453	0.508	0.675	0.108	0.025
Low	444.2	10.3	9.8	117.1	307.0	23.2	31.2	5.4	4.9	17.9	99.2	887.4	215.2	672.2	6.9	50.0	73.0	295.5	827.5	126.3	30.4	296.2	2.9	37.0	325.1	734.7	826.2	1515.4
High	654.6	20.4	21.0	164.1	449.0	36.8	71.1	8.4	12.1	28.4	135.7	1201.0	212.8	988.2	7.9	103.5	77.1	406.8	1039.9	211.6	41.3	364.2	11.7	48.4	406.8	804.8	490.1	2340.0
Mono (*p*-value)	0.063	0.062	0.022	0.114	0.112	0.109	0.043	0.219	0.031	0.103	0.213	0.548	0.689	0.420	0.338	0.279	0.939	0.390	0.920	0.724	0.423	0.478	0.258	0.794	0.060	0.023	0.440	0.028
Low	464.0	10.2	8.8	117.1	327.9	20.1	32.6	5.0	5.2	15.1	102.0	990.6	221.0	769.6	6.1	60.2	75.7	312.2	940.2	161.5	30.5	351.4	4.9	44.6	256.5	591.5	580.9	1541.9
High	643.7	21.0	22.7	166.5	433.4	40.9	71.7	8.9	12.1	32.0	134.6	1103.5	206.2	897.2	8.9	95.1	74.3	394.3	926.5	177.2	41.8	306.8	9.9	40.6	486.9	966.8	743.6	2354.1
Lymph (*p*-value)	0.756	0.912	0.331	0.470	0.476	0.892	0.109	0.820	0.980	0.811	0.335	0.592	0.677	0.591	0.168	0.530	0.536	0.318	0.727	0.941	0.786	0.718	0.684	0.683	0.442	0.311	0.727	0.213
Low	533.9	15.7	12.3	152.3	353.6	29.1	35.2	7.3	8.4	21.8	130.4	1095.8	221.9	873.9	9.4	87.1	81.1	400.0	909.3	170.7	33.9	318.6	8.2	39.5	317.3	683.1	620.4	1690.0
High	564.1	15.0	18.3	129.5	401.2	30.9	66.3	6.5	8.5	24.3	105.2	995.2	206.5	788.7	5.5	66.9	69.3	304.8	957.0	167.3	37.7	341.2	6.4	45.7	412.2	852.2	694.1	2153.9
Plt (*p*-value)	0.046	0.367	0.053	0.359	0.027	0.617	0.023	0.717	0.204	0.606	0.371	0.125	0.195	0.130	0.316	0.060	0.277	0.289	0.150	0.166	0.167	0.221	0.230	0.575	0.758	0.223	0.368	0.015
Low	453.5	12.7	9.5	126.2	305.1	26.7	29.3	6.3	6.4	20.4	105.8	901.3	190.2	711.1	6.0	46.8	64.7	300.6	836.2	138.4	26.1	291.9	4.6	38.5	346.9	668.3	563.4	1481.0
High	645.2	18.0	21.3	155.0	451.0	33.3	73.0	7.5	10.5	25.8	129.2	1187.2	237.8	949.4	8.9	106.8	85.4	401.7	1031.2	199.6	45.6	368.5	10.0	47.0	385.0	871.2	752.9	2374.4
NLR (*p*-value)	0.028	0.017	0.015	0.021	0.093	0.080	0.038	0.056	0.016	0.107	0.034	0.108	0.707	0.045	0.958	0.244	0.781	0.292	0.221	0.174	0.508	0.432	0.091	0.386	0.856	0.854	0.374	0.085
Low	443.6	8.5	8.0	104.8	322.3	18.7	31.1	3.9	4.6	14.8	90.0	894.4	220.9	673.5	7.4	58.0	77.7	300.9	850.6	138.9	31.2	305.5	3.5	36.2	377.2	785.1	751.9	1608.3
High	655.1	22.2	22.8	176.4	433.8	41.3	71.2	9.9	12.3	31.4	144.9	1194.0	207.0	987.0	7.5	95.5	72.4	401.4	1016.9	199.1	40.5	354.8	11.0	49.3	354.7	754.4	564.5	2247.1
LMR (*p*-value)	0.303	0.075	0.043	0.165	0.611	0.203	0.136	0.183	0.058	0.235	0.234	0.648	0.686	0.525	0.458	0.537	0.993	0.474	0.575	0.693	0.416	0.611	0.402	0.759	0.544	0.079	0.265	0.259
Low	599.4	20.5	21.6	162.3	395.0	38.3	65.6	9.0	11.5	29.3	133.0	1087.1	206.5	880.6	8.5	86.7	75.1	385.3	972.0	177.8	41.6	314.2	9.2	40.4	328.5	915.2	775.3	2137.9
High	499.3	10.2	9.2	118.9	361.0	21.7	36.6	4.8	5.4	17.0	101.9	1001.3	221.4	779.9	6.4	66.8	75.0	317.0	895.5	160.2	30.1	346.2	5.4	45.0	403.4	624.3	541.0	1717.5
PLR (*p*-value)	0.002	0.101	0.019	0.008	0.004	0.084	0.002	0.119	0.155	0.091	0.013	0.055	0.149	0.052	0.335	0.075	0.158	0.112	0.117	0.191	0.173	0.378	0.251	0.722	0.969	0.670	0.886	0.035
Low	401.8	10.6	8.3	99.8	283.1	18.8	21.8	4.4	6.2	14.4	85.4	865.7	187.6	678.2	6.1	48.3	61.6	275.9	827.7	140.0	26.3	302.6	4.7	40.0	368.3	734.1	673.3	1539.9
High	697.0	20.1	22.5	181.4	472.9	41.2	80.5	9.3	10.8	31.8	149.6	1222.7	240.4	982.3	8.8	105.2	88.5	426.4	1039.8	197.9	45.4	357.8	9.9	45.4	363.6	805.4	643.0	2315.5
SII (*p*-value)	0.012	0.151	0.026	0.063	0.014	0.189	0.011	0.269	0.134	0.191	0.087	0.059	0.293	0.046	0.373	0.049	0.298	0.238	0.027	0.045	0.340	0.093	0.053	0.515	0.982	0.521	0.236	0.078
Low	429.2	11.2	8.6	111.6	297.8	21.5	26.9	5.1	6.0	16.3	95.3	868.7	194.6	674.0	6.2	45.4	65.1	295.1	784.9	124.9	29.1	277.8	3.0	37.8	367.4	716.2	782.8	1601.1
High	669.6	19.5	22.2	169.6	458.3	38.5	75.4	8.6	10.9	29.9	139.7	1219.7	233.3	986.4	8.7	108.1	85.0	407.3	1082.5	213.0	42.6	382.6	11.6	47.6	364.5	823.3	533.6	2254.3

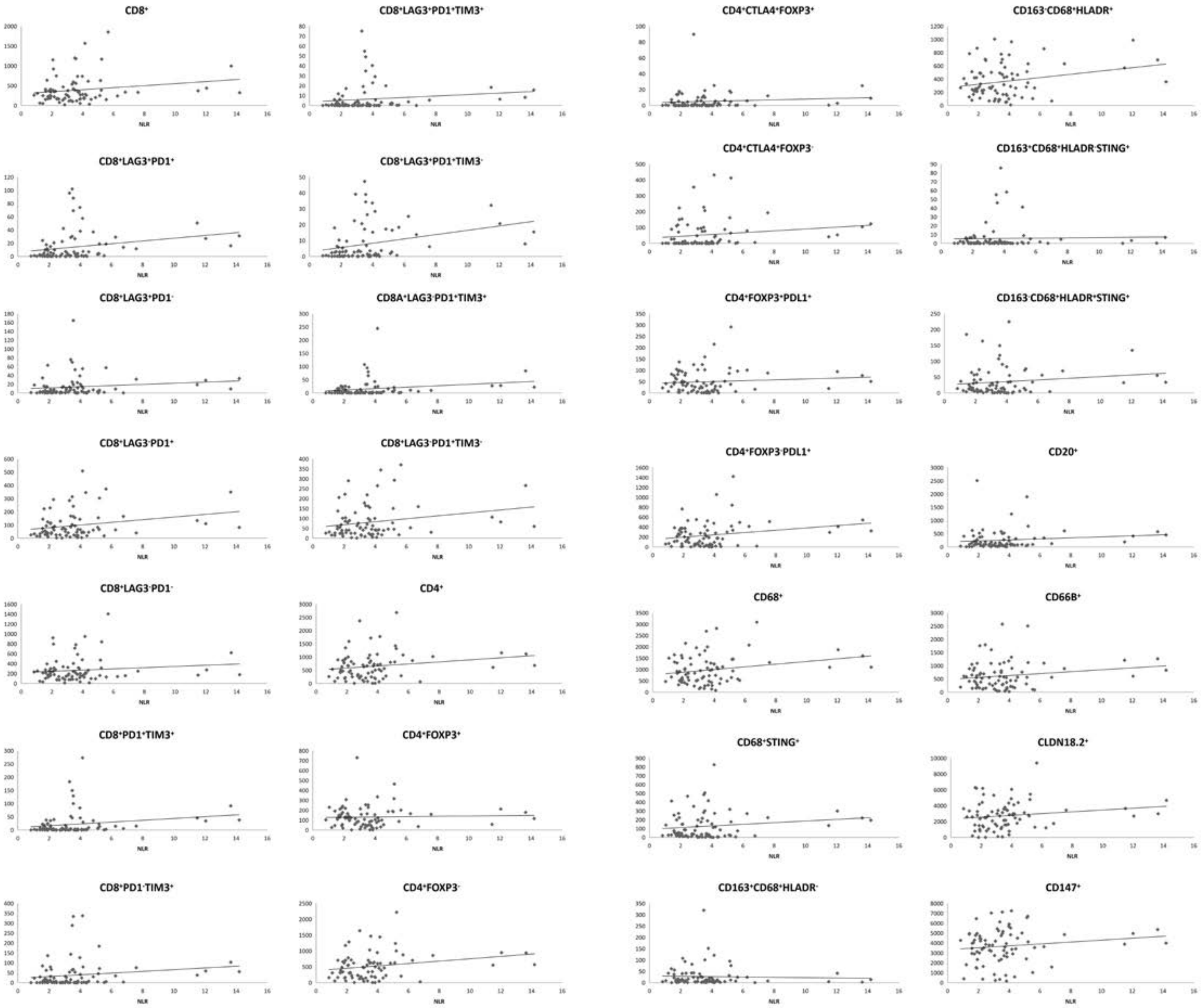

Supplementary Figure S1. The relationship of NLR with the density of immune cells in tumor core.

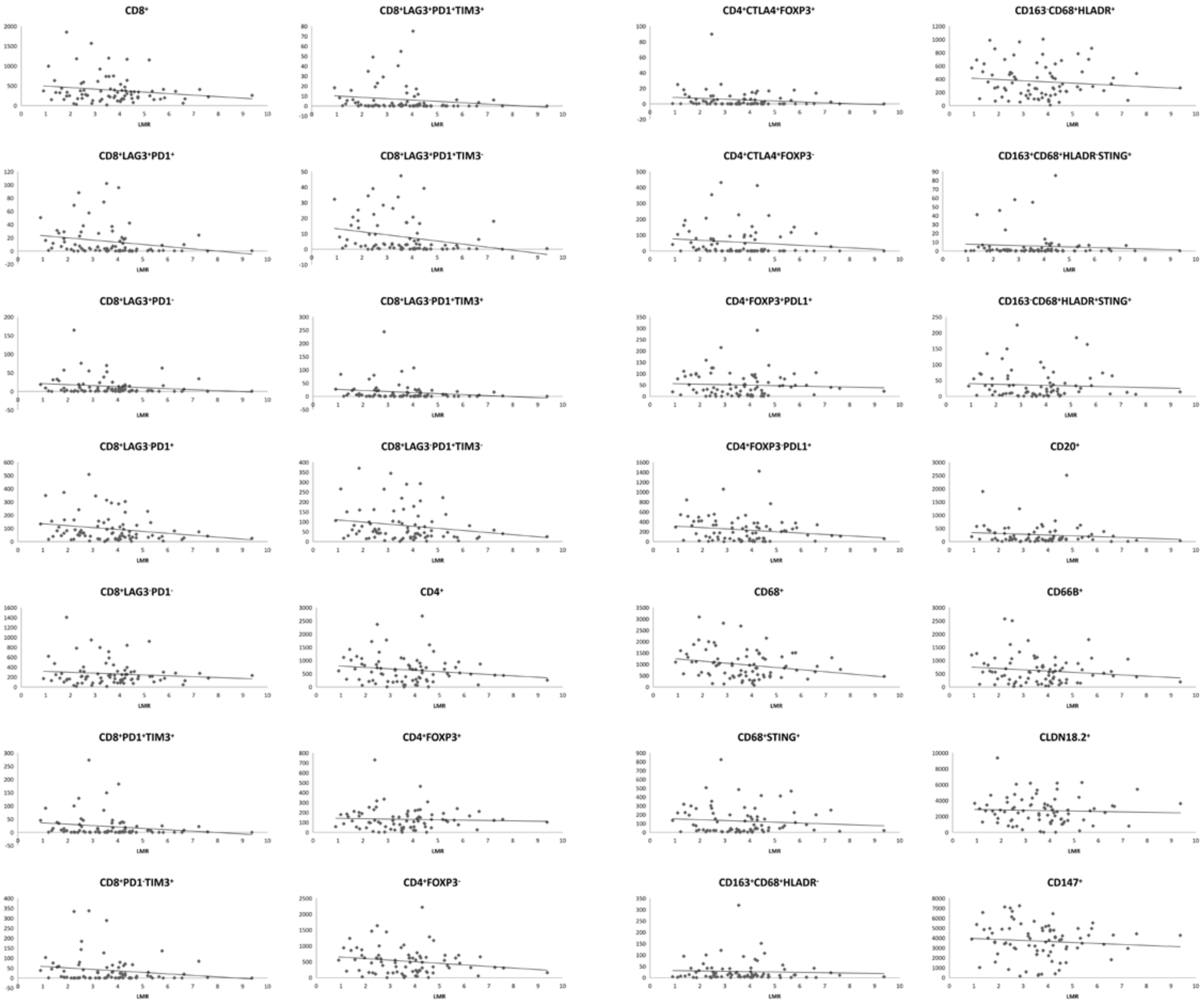

Supplementary Figure S2. The relationship of LMR with the density of immune cells in tumor core.

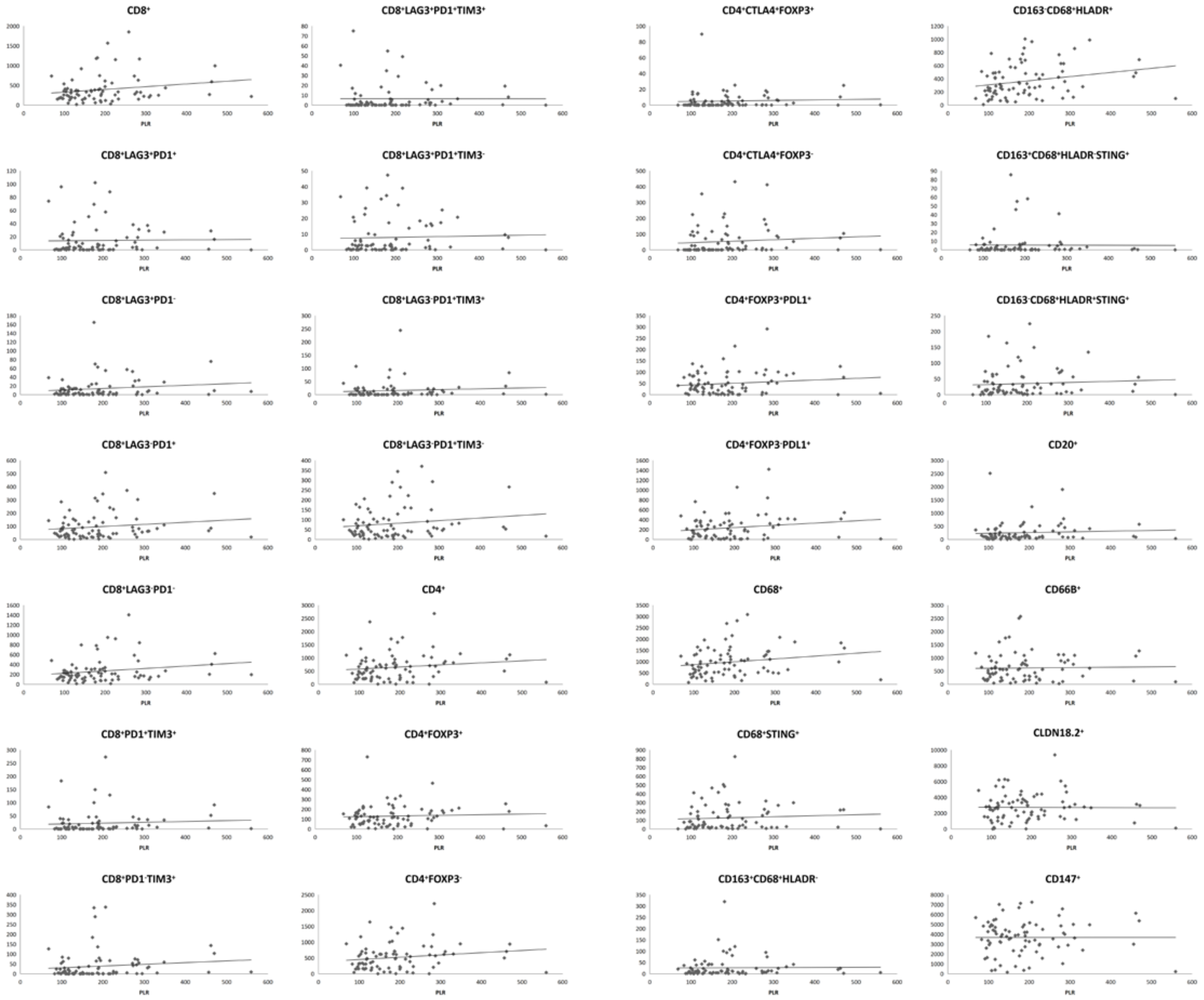

Supplementary Figure S3. The relationship of PLR with the density of immune cells in tumor core.

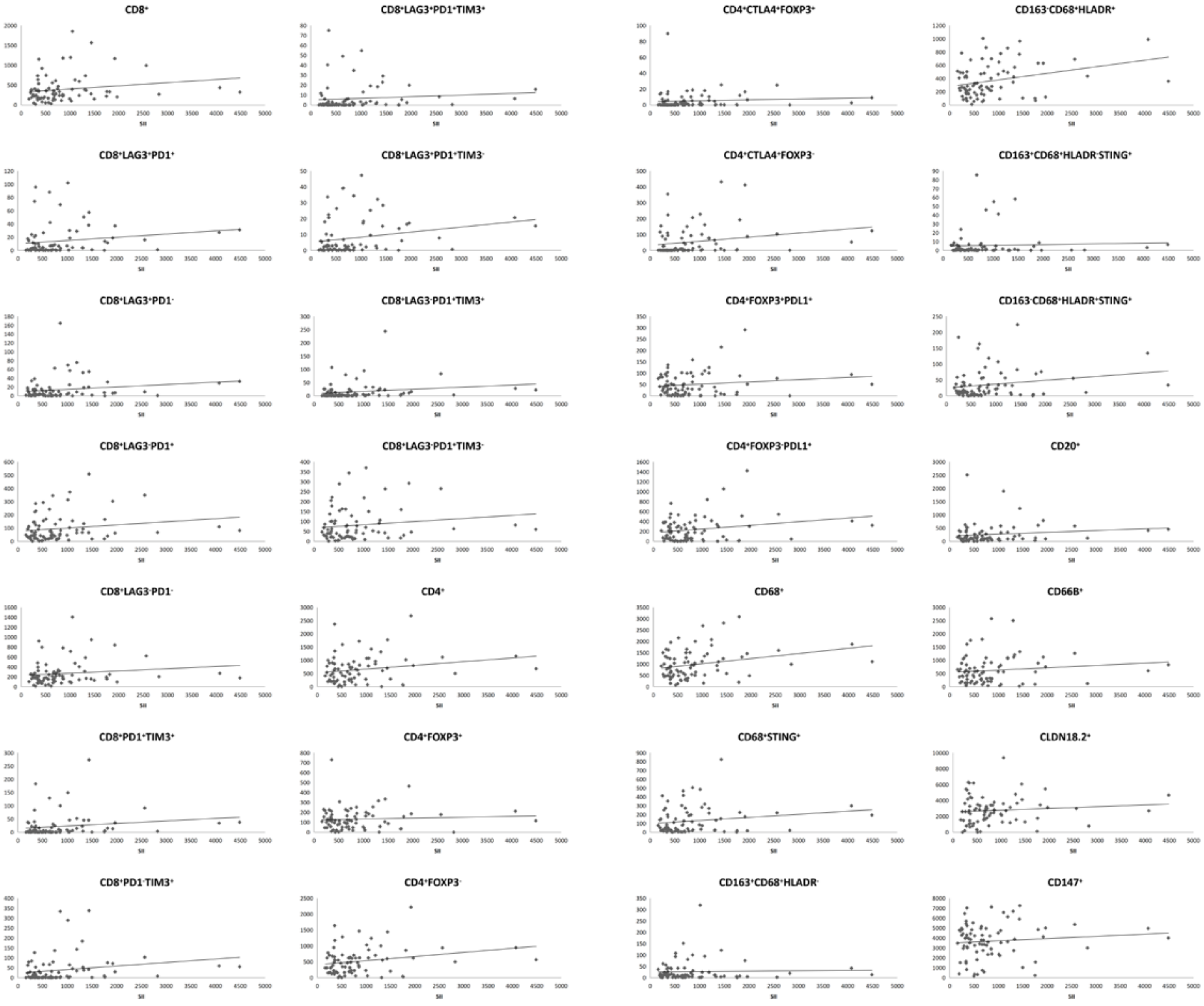

Supplementary Figure S4. The relationship of SII with the density of immune cells in tumor core.

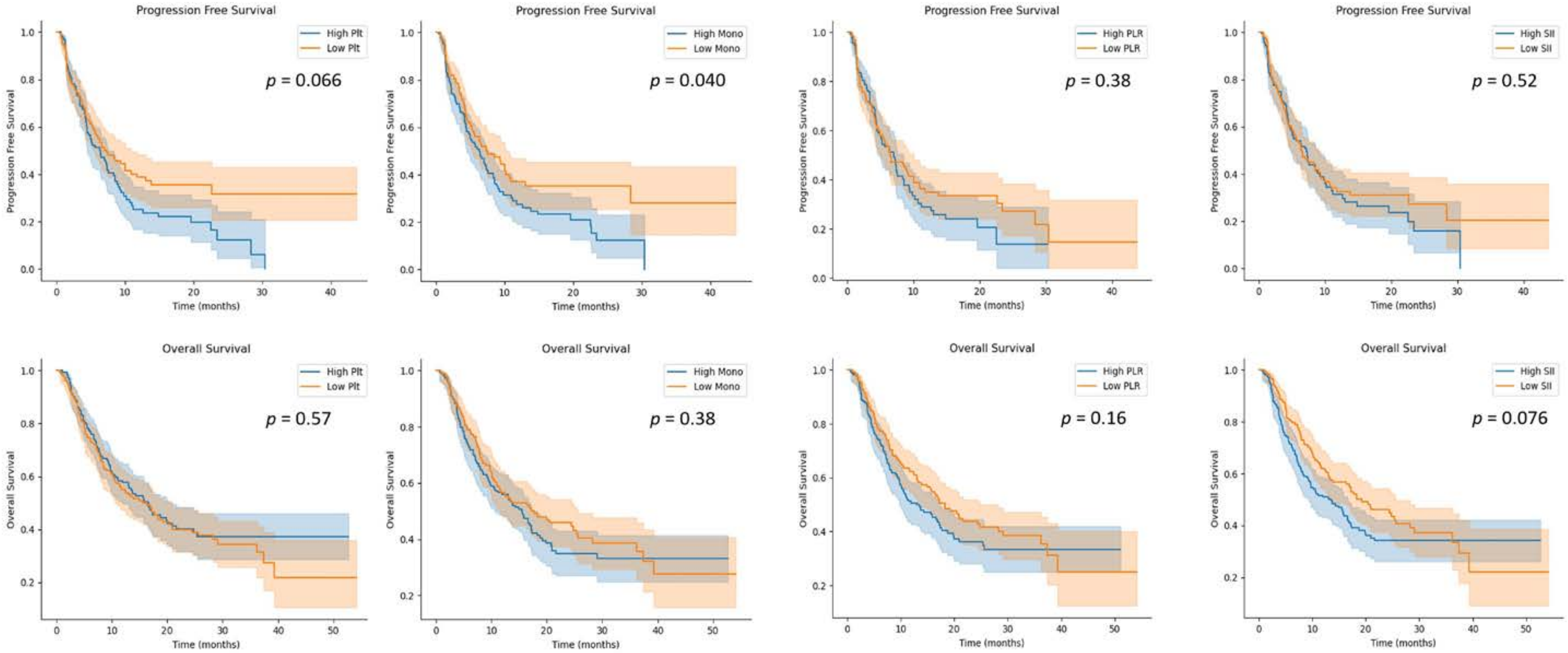

Supplementary Figure S5. Kaplan-Meier curves for progression-free survival and overall survival according to blood indexes (Plt, Mono, PLR, SII).
